# Factors associated with diagnostic delay and prognosis in hospitalized patients with strongyloidiasis in Okinawa, Japan

**DOI:** 10.1371/journal.pntd.0013380

**Published:** 2025-08-06

**Authors:** Seishiro Arima, Naoya Nishiyama, Yuri Higure, Yui Naha, Teruhito Uchihara, Shuhei Ideguchi, Hideta Nakamura, Kazuya Miyagi, Masashi Nakamatsu, Shusaku Haranaga, Takeshi Kinjo, Akihiro Tokushige, Morikazu Akamine, Kazuko Yamamoto

**Affiliations:** 1 Department of Respiratory Medicine, Okinawa Red Cross Hospital, Naha, Okinawa, Japan; 2 First Department of Internal Medicine, Division of Infectious, Respiratory, and Digestive Medicine, University of the Ryukyus Graduate School of Medicine, Ginowan, Okinawa, Japan; 3 Comprehensive Health Professions Education Center, University Hospital, University of the Ryukyus, Ginowan, Okinawa, Japan; 4 Department of Clinical Pharmacology and Therapeutics, University of the Ryukyus Graduate School of Medicine, Ginowan, Okinawa, Japan; National Institutes of Allergy and Infectious Diseases, NIH, UNITED STATES OF AMERICA

## Abstract

**Background:**

Prognosis and factors correlated to diagnostic delays in patients with strongyloidiasis, a parasitic disease, remain poorly understood. This study investigated the relationship among diagnostic delay, prognosis, and eosinophilia in patients with strongyloidiasis.

**Methodology/Principal findings:**

Hospitalized patients with a confirmed diagnosis of strongyloidiasis at a single hospital over 10 years (2013–2023) were retrospectively screened. Fifty-five inpatients were included (median age, 81 years); 34 patients (61.8%) were male, and all but one patient was born in Okinawa before 1960. Duration from onset of symptoms to diagnosis was 10 days (median), hospitalization length was 37 days (median), and eight patients (14.5%) died. We classified diagnoses made after ≥30 days as delayed diagnosis and compared the patients with and without delayed diagnosis. Patients with delayed diagnosis had more in-hospital deaths than their counterparts (55.6% versus [vs.] 7.1%, **P* *< 0.005). Compared with patients diagnosed earlier, those with delayed diagnosis were also characterized by older age (90 vs. 78.5 years, *P* < 0.005), more frequent fever (55.6% vs. 19.6%, *P* = 0.037), lower hemoglobin levels (10.1 vs. 11.8 g/dL, *P* = 0.0363), absence of eosinophilia (0% vs. 22%, *P* = 0.015), higher rates of sepsis (50% vs. 10.7%, *P* = 0.03), and prolonged hospitalization (77 vs. 23.5 days, *P* < 0.005). Diagnostic delay even after adjusting for age and sex using multivariate logistic regression analyses was a significant risk factor for mortality (odds ratio = 11.3, *P* = 0.022). Patients without eosinophilia were older than those with eosinophilia (84.5 vs. 76 years, *P* = 0.005) and not associated with in-hospital death (23.3% vs. 4.8%, **P* *= 0.118).

**Conclusions/Significance:**

Diagnostic delays are associated with a poor prognosis of strongyloidiasis. The absence of eosinophilia led to overlooked diagnoses. Screening should be considered before starting immunosuppressive therapy; relying on eosinophil counts could delay diagnosis.

## Author summary

Strongyloidiasis, a parasitic disease transmitted percutaneously from soil, mainly affects the small intestine. Diagnostic delays may worsen prognosis because the disease can become severe in immunosuppressed hosts, resulting in disseminated strongyloidiasis or hyperinfection. However, few studies have investigated the diagnostic delay associated with strongyloidiasis. This study examined the relationship between diagnostic delay and strongyloidiasis prognosis as well as factors associated with diagnostic delay at a single general hospital in Okinawa Prefecture, Japan. Fifty-five inpatients with strongyloidiasis were included from 2013 to 2023.

Patients with strongyloidiasis diagnosed in ≥30 days showed more frequent in-hospital deaths (55.6% versus [vs.] 19.6%, *P* = 0.037), more frequent sepsis, and longer hospital stays with the characteristics of older age (median age: 86.5), anemia, and normal eosinophil counts compared with those diagnosed in <30 days. Patients without eosinophilia were older than those with eosinophilia (76 vs. 84.5 years, **P* *= 0.005). Strongyloidiasis should be considered in older individuals from endemic areas even if blood eosinophil counts are normal. In Japan, patients born before 1960 are screened for strongyloidiasis.

## Introduction

Strongyloidiasis, caused by the nematode, *Strongyloides stercoralis,* is transmitted percutaneously from soil and primarily affects the small intestine; it is distributed globally but is particularly prevalent in tropical and subtropical regions, with an estimated 30–100 million people infected worldwide [1]. Strongyloidiasis may present with cutaneous or gastrointestinal symptoms; however, it is asymptomatic in over 60% of cases and is only indicated by an increased blood eosinophil count [1]. Symptoms are usually mild and nonspecific, including nausea, vomiting, diarrhea, constipation, epigastric pain, weight loss, and skin symptoms [1]. Immunosuppressive therapy leads to uncontrolled *Strongyloides* reproduction and a large increase in larvae, resulting in the development of disseminated strongyloidiasis and hyperinfection syndrome [2]. Co-infection with human T-cell leukemia virus type 1 (HTLV-1) is common and has been associated with the development of severe strongyloidiasis. Such co-infection may lead to more aggressive disease courses and resistance to standard treatment, as highlighted in a recent disease review [2], and the fatality rate of disseminated strongyloidiasis is very high, with 68.5% of patients having fatal outcomes according to a recent systematic review [3]. The literature also points out the delay in diagnosis and mentions that it is imperative to treat the disease in the chronic phase before disseminated strongyloidiasis or hyperinfection syndrome develops [3].

There are various methods of testing for strongyloidiasis, but the primary methods are stool examination and serological testing. Stool tests include direct smear, Kato-Katz technique, formalin-ether precipitation, Baermann sedimentation, and agar plate culture [4]. Agar plate culture is the most sensitive, but sensitivity decreases when larval shedding is low [5]. Real-time polymerase chain reaction with stool specimens is another method [6], but it is not commonly used in Japan because it is not covered by insurance. Serological tests are performed to detect antibodies against *S. stercoralis*. The sensitivity is high, ranging from 73% to 100%, but the specificity is low due to cross-reactivity with other nematodes [7]. Additionally, sensitivity is reduced in immunocompromised states [5]. For diagnosis, it is important to combine testing methods while taking the patient’s background characteristics into consideration. Serological tests, however, are not covered by insurance in Japan and must be paid for out of pocket, and they are often performed when strongyloidiasis is suspected despite the absence of worms in stool tests (agar plate culture method). Since such patients are often immunocompromised, the agar plate culture method is commonly used in Japan. In Japan, strongyloidiasis is more common in the Okinawa and Kyushu regions than in other regions and is prevalent among older adults [8]. Okinawa Prefecture is a subtropical area that provides a favorable environment for *S. stercoralis* owing to its temperature and humidity. According to a 2016 report, the prevalence of *S. stercoralis* infection in Okinawa Prefecture was 5.2% and was more common among those born before 1960 than among those born after 1960 [9]. *S. stercoralis* maintains its infection for several decades within the same host through a unique life cycle. The infective filariform larvae enter the skin and reach the lungs in the circulation. The larvae migrate through the alveoli, ascend the trachea, and are coughed up and swallowed into the esophagus. From there, larvae travel to the small intestine where they mature into parasitic female adults. The parasitic females burrow into the lining of the gut and produce eggs and hatch in the mucosa. This unique life cycle called autoinfection demonstrates the remarkable persistence of *S. stercoralis* [10]. Owing to the long duration of infection, the disease can develop after the host migrates from an endemic to a non-endemic area [11,12].

Improved public health has reduced the opportunities for health care providers to diagnose strongyloidiasis in recent years, which may have led to some cases being missed and to a lack of awareness in non-endemic regions. Even if physicians recognize strongyloidiasis, their experience in caring for patients with strongyloidiasis has been decreasing. Delayed diagnosis may contribute to severe outcomes [11,12]; however, the factors associated with diagnostic delays are not well understood. In this study, we examined the relationship between diagnostic delay and strongyloidiasis prognosis, as well as the factors associated with diagnostic delay, at a general hospital in Okinawa, Japan.

## Materials and methods

### Ethics statement

This study was conducted retrospectively by analyzing previously collected data. Based on ethical guidelines, individual consent was not obtained, and an opt-out method was implemented instead. Study details, including the purpose and data usage, were disclosed publicly, allowing participants to decline participation within a specified period. The study protocol was approved by the Ethics Committee of Okinawa Red Cross Hospital (approval number: 2023–4).

### Patients

**Fig 1:**
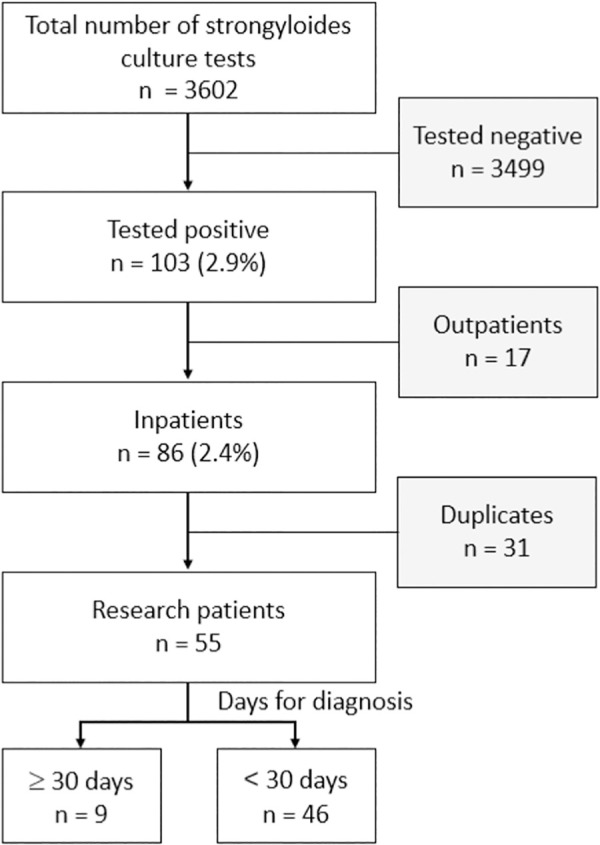
Study participants from Okinawa Red Cross Hospital (2013/4–2023/3).

Okinawa Red Cross Hospital (ORCH) is an acute care hospital located in Naha City (population: 300,000) that annually diagnoses strongyloidiasis. Among the patients with a positive *S. stercoralis* culture test and a confirmed diagnosis of strongyloidiasis at the ORCH from April 2013 to March 2023 for whom electronic medical records were available, we included those who were hospitalized and excluded duplicate cases (Fig 1). Patient characteristics (age, sex, place of birth, and underlying diseases), specimen isolation, symptoms at admission, laboratory test results on the day of symptom onset, days required for diagnosis (number of days from symptom onset and findings to the date of positive *S. stercoralis* culture), length of hospitalization, treatment, and outcomes were investigated retrospectively. In asymptomatic patients, laboratory results were obtained at admission and the number of days required for diagnosis was counted from admission to diagnosis. Additionally, records of blood culture submissions and results during hospitalization were collected to determine whether the patients had sepsis with bacteremia.

### Diagnosis of strongyloidiasis

The examination for strongyloidiasis is performed in our hospital using the ordinary agar plate culture method [13]. A stool specimen of digitus minimus size (2 g) was placed on an agar plate medium (Müller-Hinton S agar medium; Eiken, Japan) and incubated at room temperature for 2 days. In all cases where rutted bacterial colonies were observed on agar plates, microscopic examination confirmed the presence of worms. If the worms were considered to be *S. stercoralis* larvae, the sample was considered positive for *S. stercoralis*. Patients who tested positive for strongyloidiasis were identified from clinical laboratory records and enrolled in this study. On the basis of the patient’s history and the symptoms, we diagnosed patients with acute infection, chronic infection with symptoms, or chronic infection without symptoms.

### Outcomes

Strongyloidiasis and hyperinfection syndrome have a short onset time, with diagnoses established after a median of 1.5 months (approximately 45 days) of symptoms [14]. A shorter diagnostic duration is necessary to prevent the development of severe infection. Therefore, we defined delayed diagnosis in strongyloidiasis as requiring 30 days from symptom onset to diagnosis to prevent the development of severe infection. We then compared mortality as the primary outcome between the early and delayed diagnosis groups. As the secondary analysis, we examined the factors associated with the diagnostic delay. Subsequently, we divided the patients into two groups: the deceased group (patients who experienced in-hospital death) and the surviving group to assess the time to diagnosis and the factors associated with mortality. We also hypothesized that the presence or absence of hypereosinophilia may be related to a delayed diagnosis. Thus, we divided the patients into groups based on the presence or absence of hypereosinophilia to investigate their association with delayed diagnosis and examined the characteristics of fecal nematodes in patients without hypereosinophilia.

### Statistical analysis

Continuous variables were analyzed for statistical significance using the Student t-test and the Wilcoxon test, and nominal variables were analyzed for statistical significance using the Fisher exact test. We also conducted logistic regression analyses to assess the associations between risk factors and mortality. Crude odds ratios (ORs) with 95% confidence intervals were calculated using univariate models. Multivariate logistic regression was performed to adjust for confounders, including age, sex, and diagnostic delay, with adjusted ORs (aORs) reported. Continuous variables, such as age, were included without categorization. Statistical significance was determined using two-tailed tests, with an alpha level of 0.05. Statistical analyses were performed using JMP 13 (SAS Institute Inc., Cary, NC, USA).

## Results

Table 1 shows the characteristics of the patients, their laboratory findings at diagnosis, and a comparison of characteristics with the groups of patients who had a diagnostic delay or were diagnosed in less than 30 days of hospitalization. Throughout the study period, 103 patients (2.9%) tested positive out of 3,605 *Strongyloides* culture tests performed according to the physicians’ decisions. Overall, 55 of the 84 inpatients were included in the study. The median age of included patients was 81 years, with 34 males and 21 females, all of whom were Japanese and born in Okinawa Prefecture except for one Vietnamese person. The underlying conditions were malignancies in 20 patients (36.4%), history of chemotherapy for malignancies in 6 patients (10.9%), anti-HTLV-1 antibody positivity in 10 patients (33.3%), immunosuppressive therapy in 12 patients (21.8%), and steroid use in 7 patients (12.7%). There were no cases of acute infection, 51 cases of chronic infection, and 4 cases of asymptomatic positive result. The most common symptoms on admission were gastrointestinal symptoms (34.5%) with abdominal pain, loss of appetite, vomiting, diarrhea, bloody stools, abdominal swelling, and fever in 14 patients (25.5%), followed by respiratory symptoms (16.4%), disturbance of consciousness (5.5%), skin symptoms (3.6%), and others (leg edema and chest symptoms). Four patients (7.3%) were asymptomatic, three were admitted for anti-cancer chemotherapy and one for anti-tuberculosis treatment.

**Table 1:** Patients’ characteristics.

Patient Characteristic	Overall (n = 55)	Diagnosis ≥30 days (n = 9)	Diagnosis <30 days (n = 46)	*P*-value
**Age (years)**	81 (24–94)	90.0 (81–94)	78.5 (24–93)	<0.005
**Male sex**	34 (61.8)	5 (55.6)	29 (63.0)	0.719
**Born in Okinawa**	54 (98.2)	9 (100)	45 (97.8)	1
**Malignancy**	20 (36.4)	3 (33.3)	17 (37.0)	1
**Anti-cancer chemotherapy**	6 (10.9)	0 (0)	6 (13.0)	0.574
**Immunosuppressive therapy**	12 (21.8)	1 (11.1)	11 (23.9)	0.666
**Steroid use**	7 (12.7)	1 (11.1)	6 (13.0)	1
**Anti-HTLV-1 antibody positive**^a^	10 (33.3)	0 (0)	10 (38.5) ^g^	0.272
**Asthma**	5 (9.1)	0 (0)	5 (10.9)	0.577
**Symptom**				
**Gastrointestinal**	19 (34.5)	4 (44.4)	15 (32.6)	0.703
**Fever**	14 (25.5)	5 (55.6)	9 (19.6)	0.037
**Respiratory**	9 (16.4)	1 (11.1)	8 (17.4)	1
**None**	4 (7.3)	0 (0)	4 (8.7)	1
**Impaired consciousness**	3 (5.5)	0 (0)	3 (6.5)	1
**Skin symptoms**	2 (3.6)	0 (0)	2 (4.4)	1
**Others**	4 (7.3)	0 (0)	4 (8.7)	1
**Laboratory finding**				
**WBC (/**μ**L)**	8400 (1900–33000)	7600 (1900–13900)	9050 (1900–33000)	0.083
**Neutrophil (/**μ**L)**^**b**^	5888 (273.6–24002)	4438 (274–12037)	5958 (1136–24002)	0.381
**Lymphocyte (/**μ**L)**^**c**^	1178 (210.9–4794.6)	969.85 (403–1749)	1418 (211–4795)	0.129
**Eosinophil (/**μ**L)**^**d**^	462 (0–6762)	103 (21–169)	484 (0–6762)	0.028
**Eosinophilia (>500/**μ**L)**^**e**^	22 (41.2)	0 (0)	22 (50)^h^	0.015
**Hb (g/dL)**	11.7 (7.1–16.4)	10.1 (7.2–12.8)	11.8 (7.1–16.4)	0.036
**CRP (mg/dL)^f^**	2.4 (0.04–19.2)	5 (0.3–12.6)	2.2 (0.04–19.2)	0.235

^a^ HTLV-1, human T-cell leukemia virus type 1 (n = 30);

^b^,

^d^,

^e^ n = 52;

^c^ n = 51 (In one case, lymphocytes were not measurable in ATL.);

^f^ n = 50;

^g^ n = 26;

^h^ n = 44. Abbreviations: WBC, white blood cell; Hb, hemoglobin; CRP, C-reactive protein; ATL, adult T-cell leukemia. Continuous variables are expressed as median (range), and nominal variables are expressed as number (%).

Frequent laboratory findings at the time of diagnosis included peripheral blood eosinophilia (> 500/μL; 22/52, 42.3%) and seven positive blood cultures (17.9%) for *Escherichia coli* (n = 2), *Bacillus* spp. (n = 2), *Pseudomonas aeruginosa* (n = 1), *Klebsiella pneumonia* (n = 1), and co-infection with *K. pneumoniae* and *Candida* spp. (n = 1). *S. stercoralis* was isolated in 53 patients (96.4%) from stool and two patients (3.6%) from duodenal fluid. The median duration of diagnosis was 10 days (range: 0–83), and the median hospitalization length was 37 days (range: 3–183). The median duration from diagnosis to treatment was 3 days (range: 0–69). One patient, who took 69 days to treat, was positive on initial testing but was not diagnosed by the attending physician. Two months later, the patient was diagnosed and treated when he was retested for eosinophilia and tested positive. In this case, the delay in treatment did not cause any disadvantage. Fifty patients (90.9%) received ivermectin during hospitalization and eight patients (14.5%) died; the cause of death was attributed to sepsis in four patients and pneumonia, acute respiratory distress syndromes, strangulated ileus, and stroke in one patient each. No patients were diagnosed with disseminated strongyloidiasis or hyperinfection syndrome at the time of clinical diagnosis.

In the comparison of patients with diagnostic delay and others, nine patients whose strongyloidiasis diagnosis required ≥30 days were significantly older (median: 90.0 versus [vs.] 78.5, **P* *< 0.005). Underlying malignancies, chemotherapy, immunosuppressive therapy, and steroid use were not associated with diagnostic delays. Patients with diagnostic delay showed fever (55.6% vs. 19.6%, **P* *= 0.037), lower Hb (10.1 vs. 11.8 g/dL, **P* *= 0.0363), and normal eosinophil counts (0% vs. 22%, **P* *= 0.015) compared with those without.

Table 2 shows the outcomes of the patients who had a diagnostic delay or were diagnosed in less than 30 days of hospitalization. Patients with diagnostic delays had more frequent sepsis (50% vs. 10.7%, *P* = 0.03), in-hospital deaths (55.6% vs. 7.1%, **P* *< 0.005) and longer hospitalization length (77 vs. 23.5 days, *P* < 0.0005) than those without.

**Table 2:** Outcomes.

Characteristic (n = 55)	Overall (n = 55)	Diagnosis ≥30 days (n = 9)	Diagnosis <30 days (n = 46)	*P*-value
**In-hospital death**	8 (14.5)	5 (55.6)	3 (6.5)	<0.005
**Sepsis with bacteremia** ^**a**^	7 (19.4)	4 (50.0)^b^	3 (10.7)^c^	0.03
**Hospitalization length**	37 (3–183)	77 (34–183)	23.5 (3–120)	<0.005

^a^ n = 36,

^b^ n = 8,

^c^ n = 28; blood cultures collected during hospitalization. Continuous variables are expressed as median (range), and nominal variables are expressed as number (%).

Table 3 shows comparison of the characteristics of the deceased and surviving patients. The deceased group was significantly older than the surviving group (86.5 vs. 79.0 y, *P* = 0.036), with no sex differences found between the groups. Malignancies were more frequently observed in the deceased group than in the surviving group (62.5% and 31.9%, respectively, **P* *= 0.124), without a significant difference. Gastrointestinal symptoms were more common in the deceased group than in the surviving group (75% vs. 27.7%; **P* *= 0.0156), as were sepsis and positive blood cultures (50% vs. 6.4%, **P* *= 0.016), significantly longer duration of strongyloidiasis (32.5 vs. 9 days, **P* *= 0.032), and longer hospitalization (65 vs. 24 days, **P* *= 0.024).

**Table 3:** Comparison of the characteristics of the deceased and surviving patients.

Patient Characteristic	Deceased (n = 8)	Survived (n = 47)	*P*-value
**Age (years)**	86.5 (75–94)	79 (24–93)	0.036
**Male sex**	6 (75.0)	28 (59.6)	0.696
**Born in Okinawa**	8 (100)	46 (97.9)	1
**Malignancy**	5 (62.5)	15 (31.9)	0.124
**Chemotherapy**	0	6 (12.8)	0.577
**Immunosuppressive therapy**	2 (25.0)	10 (21.3)	1
**Steroid use**	2 (25.0)	5 (10.6)	0.267
**Anti-HTLV-1 antibody positive**^a^	1 (20.0)	9 (36.0)	0.64
**Asthma**	0	5 (10.6)	1
**Symptom**			
**Gastrointestinal**	6 (75.0)	13 (27.7)	0.015
**Fever**	1 (12.5)	13 (27.7)	0.663
**Respiratory**	2 (25.0)	7 (14.89)	0.604
**None**	0	4 (8.5)	1
**Impaired consciousness**	0	3 (6.4)	1
**Skin symptoms**	0	2 (4.3)	1
**Others**	0	4 (8.5)	1
**Laboratory finding**			
**WBC (/μL)**	6400 (2900–33000)	9100 (1900–27400)	0.242
**Neutrophil (/μL)**^**b**^	4159.2 (2131.2–13959)	6200 (273.6–24002.4)	0.501
**Lymphocyte (/μL)**^**c**^	1178.1 (388.8–2259.6)	1255.1 (210.9–4794.6)	0.794
**Eosinophil (/μL)**^**d**^	128.6 (0–1529)	400.5 (0–6762)	0.145
**Eosinophilia (>500/μL)**^**e**^	1 (12.5)	21 (47.7)	0.117
**Hb (g/dL)**	11.3 (7.2–14.5)	11.7 (7.1–16.4)	0.542
**CRP (mg/dL)**^**f**^	3.27 (0.26–16.12)	2.26 (0.004–19.21)	0.551
**Specimen of the culture for *Strongyloides***			
**Stool**	8 (100)	45 (95.7)	1
**Duodenal fluids**	0	2 (4.3)	1
**Clinical course**			
**Blood culture positive**^**g**^	4 (50.0)	3 (6.4)	0.016
**Hospitalization length**	24 (3–121)	65 (34–183)	0.024
**Ivermectin treatment**	7 (87.5)	43 (91.5)	0.559

^a^ HTLV-1, human T-cell leukemia virus type 1 (n = 30);

^b^,

^d^,

^e^ n = 52;

^c^ n = 51;

^f^ n = 50;

^g^ n = 36; blood cultures collected during hospitalization. Abbreviations: WBC, white blood cell; Hb, hemoglobin CRP, C-reactive protein. Continuous variables are expressed as median (range), and nominal variables are expressed as number (%).

Table 4 presents the results of univariate and multivariate logistic regression analyses, showing ORs for mortality with age, sex, and diagnostic delay as variables. Among these, diagnostic delay was the only variable that demonstrated statistical significance (aOR = 11.3, *P*-value = 0.022).

**Table 4:** Unadjusted and adjusted odds ratios for mortality.

Characteristic (n = 55)	Deceased n = 8 (%)	Survived n = 47 (%)	Unadjusted OR (95% Cl)	*P*-value	Adjusted OR (95% CI)	*P*-value
**Days for diagnosis** ≥**30 days**	5 (62.5)	4 (8.5)	17.9 (3.08–104.2)	0.0016	11.3 (1.4–135.6)	0.0222
**Age**	86.5 (75–94)	79 (24–93)	1.16 (1.03–13.4)	0.0074	1.08 (0.96–1.27)	0.2666
**Male sex**	6 (75)	28 (59.6)	2.04 (0.37–11.2)	0.696	3.5 (0.53–38.8)	0.2

OR, odds ratio; CI, confidence interval. Univariate analyses provided the crude ORs for each variable. Multivariate analyses were adjusted for age, sex, and diagnostic delay. Continuous variables, including age, were entered as continuous predictors in the model. ORs for age represent the change in odds per 1-year increase. Statistical significance was defined as a *P*-value of <0.05.

Table 5 shows the relationship between the patient characteristics and eosinophilia. Patients without eosinophilia were approximately 10 years older than those with eosinophilia (84.5 vs. 76 years, **P* *= 0.005). Sex, immunosuppressive factors, and symptoms did not differ between the groups. Longer hospitalizations (median: 17.5 vs. 49 days, *P* = 0.081) and frequent in-hospital deaths (23.3% vs. 4.8%, **P* *= 0.119) were observed in patients with normal eosinophil counts compared with those without, although the differences were not significant.

**Table 5:** Characteristics of patients with and without eosinophilia.

Characteristic (n = 52)	Eosinophilia (n = 22)	Without eosinophilia (n = 30)	*P*-value
**Age**	76 (24–91)	84.5 (61–94)	0.005
**Male sex**	15 (68.2)	18 (60.0)	0.575
**Born in Okinawa**	21 (95.5)	30 (100)	0.4231
**Malignancy**	8 (36.4)	10 (33.3)	1
**Chemotherapy**	2 (9.1)	3 (10)	1
**Immunosuppressive therapy**	4 (18.2)	7 (23.3)	0.741
**Steroid use**	2 (9.5)	5 (16.7)	0.685
**Anti-HTLV-1 antibody positive**^a^	4 (18.2)	5 (16.7)	1
**Asthma**	3 (13.6)	2 (6.7)	0.6391
**Symptom**			
**Gastrointestinal**	6 (27.3)	12 (40)	0.3898
**Fever**	3 (13.6)	10 (33.3)	0.1938
**Respiratory**	6 (27.3)	3 (10)	0.1441
**None**	2 (9.1)	2 (6.7)	1
**Impaired consciousness**	0	3 (10)	0.2534
**Skin symptoms**	1 (4.6)	0	0.4231
**Others**	2 (9.1)	2 (6.7)	1
**Hospitalization length**	17.5 (3–120)	49 (5–183)	0.0815
**In-hospital death**	1 (4.8)	7 (23.3)	0.119

^a^ HTLV-1, human T-cell leukemia virus type 1 (n = 29). Continuous variables are expressed as median (range), and nominal variables are expressed as number (%).

**Fig 2:**
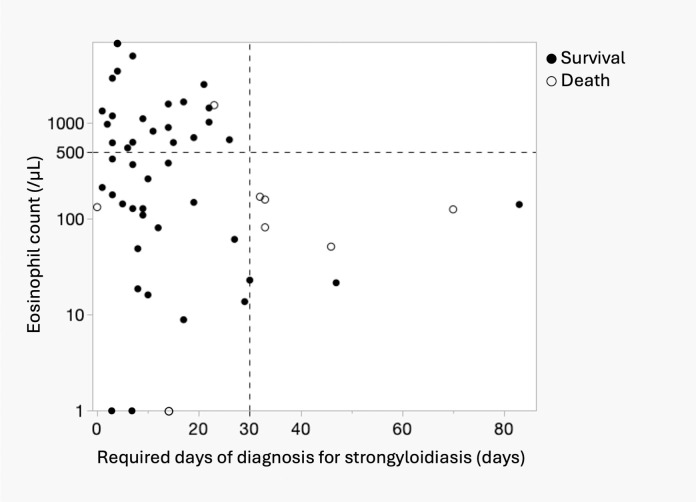
Relationship between the number of days required to diagnose strongyloidiasis and the peripheral eosinophil count. The graph area is divided into four by the required days of diagnosis on the X-axis and eosinophil counts on the Y-axis.

Fig 2 shows the relationship between the number of days required for the diagnosis of strongyloidiasis and peripheral eosinophil counts in 52 patients, except for 3 patients for whom eosinophil counts were not assessed. Overall, three patients had zero eosinophil counts, as shown on the X-axis of Fig 2. The group diagnosed in <30 days without eosinophilia and the group without eosinophilia were the same (22/52, 42.3%), followed by the group of patients diagnosed at ≥30 days without eosinophilia (7/52, 13.4%). No patients with eosinophilia were diagnosed after 30 days. Seven of the eight patients who died in the hospital did not have eosinophilia. One patient who died on day 0 of diagnosis was screened for strongyloidiasis due to adult T-cell leukemia and died of a stroke.

## Discussion

Our study of 55 patients with strongyloidiasis showed that in-hospital deaths occurred more frequently in patients with delayed diagnosis than in those without, suggesting that diagnostic delays are associated with a poor prognosis. Additionally, three patients who died of sepsis experienced diagnostic delays, suggesting that such delays may have allowed the development of disseminated strongyloidiasis. This finding is consistent with those of previous reports; for example, a study from Central Australia reported how delays in the recognition and management of *S. stercoralis* led to increased morbidity and mortality among indigenous patients [15]. The mortality rate of severe strongyloidiasis and translocated bacterial sepsis is reported to be 87% [16–19], which is significantly higher than that of general sepsis [20]. Implementing a comprehensive screening strategy is essential for preventing diagnostic delays and improving patient outcomes.

Eosinophilia plays an important role in helminth infections by activating Th2 lymphocytes, with eosinophils acting as antigen-presenting cells in the Th2 response [21–24]. In patients with strongyloidiasis, lower peripheral eosinophil counts are associated with treatment resistance [25] and disease severity, whereas eosinophilia generally correlates with a better prognosis [12,26]. Our study population had a low eosinophilia rate (42.3%), lower than that in previous reports (50–75%) [12,27], likely due to the older age of our patients. Age-related trends indicate that eosinophilia is the highest in young individuals (2–4 years) and decreases in older patients [28], making eosinophil-based screening less effective with increasing age. Additionally, because many at-risk individuals in Japan are older and born in the southern region before 1960 [9], relying on the eosinophil count alone as a diagnostic criterion is unreliable. Eosinophil-based screening strategies for strongyloidiasis need to be carefully considered, particularly in older adults, because diagnostic delays are associated with normal eosinophil counts (Fig 2).

Patients with strongyloidiasis are frequently co-infected with HTLV-1 [2]; carriers may not develop eosinophilia during strongyloidiasis [1]. Co-infection with HTLV-1 reduces interleukin-5 and immunoglobulin E responses and switches from Th2 to Th1 responses in patients with strongyloidiasis [26]. In our study, no significant differences were observed in peripheral eosinophil levels (254.9 vs. 380.7/μL, *P* = 0.981) with or without HTLV-1 co-infection, respectively, although anti-HTLV-1 antibodies were only examined in approximately half (30) of the 55 cases.

Early diagnosis and prompt detection of strongyloidiasis in patients with normal eosinophil counts are essential for preventing severe outcomes. However, the diagnosis of strongyloidiasis is challenging because of its mild symptoms [1,13,29]. In our study, gastrointestinal symptoms were more common in the deceased group, whereas fever and anemia were more common in the delayed diagnosis group. These non-specific symptoms can further complicate the diagnosis. It is essential to screen for strongyloidiasis in patients from endemic areas who present with unexplained symptoms or sepsis even in the absence of eosinophilia. Testing for HTLV-1 antibodies may also aid in identifying co-infections in high-risk immunocompromised or older patients, particularly those receiving steroids or chemotherapy, to prevent severe strongyloidiasis.

Regarding delays from diagnosis to treatment, there were five patients for whom no treatment was given and one patient for whom it took a long time to receive treatment. In all cases, the patient was not disadvantaged by the lack of or delay in treatment, but the attending physician was unaware of the positive test result. In two cases, the physician submitting the test and the attending physician differed (e.g., the endoscopist performed the test because strongyloidiasis was suspected at the time of endoscopy), which was believed to be a factor. The reporting system for positive tests was discussed with the laboratory department, and countermeasures were considered.

The limitations of this study are that it was retrospective and conducted in a single institution with partially missing data (such as anti-HTLV-1 antibody and blood culture results). Moreover, symptomatic and asymptomatic patients were included. Additionally, due to improved sanitation in Japan, new or acute cases of *Strongyloides* infection are now extremely rare. Consequently, this study included only patients with chronic infection, and thus could not estimate the diagnostic delay and its effects on acute infections. Given the rarity of this disease and the limited sample size—only nine cases required more than 30 days for diagnosis—further prospective studies are warranted to validate these findings and refine the diagnostic criteria. However, by analyzing more than 55 hospitalized patients with strongyloidiasis, this study provides an example of the clinical practice for testing and treating strongyloidiasis in Japan. Because most of our study population was older, this study highlighted the characteristics of strongyloidiasis in this population.

## Conclusion

Strongyloidiasis is often asymptomatic and its diagnosis frequently relies on eosinophilia. Our study highlights the risk of diagnostic delays in patients without eosinophilia, particularly in older individuals from endemic areas. Screening in these populations is essential to prevent severe outcomes, as the eosinophil count alone may not be a reliable indicator. Future research should focus on alternative biomarkers and more comprehensive screening methods, especially in high-risk older populations, to enable earlier detection and prevent severe outcomes associated with diagnostic delays.

## Supporting information

S1 File Dataset of patient characteristics. (XLSX)
